# Swept-Source Anterior Segment Optical Coherence Tomography Imaging and Quantification of Bleb Parameters in Glaucoma Filtration Surgery

**DOI:** 10.3390/bioengineering10101186

**Published:** 2023-10-13

**Authors:** Jeremy C.K. Tan, Hussameddin Muntasser, Anshoo Choudhary, Mark Batterbury, Neeru A. Vallabh

**Affiliations:** 1 St. Paul’s Eye Unit, Royal Liverpool University Hospital, Liverpool L7 8YA, UK; jeremy.c.tan@unsw.edu.au (J.C.K.T.);; 2 Faculty of Medicine and Health, University of New South Wales, Kensington, NSW 2032, Australia; 3 Department of Eye and Vision Sciences, Institute of Life Course and Medical Sciences, University of Liverpool, Liverpool L69 3BX, UK

**Keywords:** glaucoma surgery, trabeculectomy, deep sclerectomy, mitomycin-C, scleral flap, surgical technique, surgical success

## Abstract

This paper describes a technique for using swept-source anterior segment optical coherence tomography (AS-OCT) to visualize internal bleb microstructure and objectively quantify dimensions of the scleral flap and trabeculo-Descemet window (TDW) in non-penetrating glaucoma filtration surgery (GFS). This was a cross-sectional study of 107 filtering blebs of 67 patients who had undergone deep sclerectomy surgery at least 12 months prior. The mean post-operative follow-up duration was 6.5 years +/− 4.1 [standard deviation (SD)]. The maximal bleb height was significantly greater in the complete success (CS) blebs compared to the qualified success (QS) and failed (F) blebs (1.48 vs. 1.17 vs. 1.10 mm in CS vs. QS vs. F, one-way ANOVA, *p* < 0.0001). In a subcohort of deep sclerectomy blebs augmented by intraoperative Mitomycin-C, the trabeculo-Descemet window was significantly longer in the complete success compared to the qualified success group (613.7 vs. 378.1 vs. 450.8 µm in CS vs. QS vs. F, *p* = 0.004). The scleral flap length, thickness, and width were otherwise similar across the three outcome groups. The quantification of surgical parameters that influence aqueous outflow in non-penetrating GFS can help surgeons better understand the influence of these structures on aqueous outflow and improve surgical outcomes.

## 1. Introduction

Glaucoma is a neurodegenerative disease caused by progressive loss of retinal ganglion cells, with a global prevalence of 3.5% in individuals aged 40 to 80 years old [1]. The reduction of intraocular pressure (IOP) is the only known modifiable risk factor for the treatment of glaucoma [2]. This can be done using topical eye drops, laser treatment, microinvasive glaucoma surgery, or glaucoma filtration surgery (GFS). Glaucoma filtration surgery is often performed to achieve greater intraocular pressure reduction or when other earlier interventions fail to halt glaucoma progression. In GFS, an artificial passage is created for the drainage of aqueous humor from the anterior chamber of the eye into the space below the conjunctiva (the subconjunctival space) and Tenon’s capsule (subtenons), with subsequent formation of a structure known as a bleb [3]. 

Non-penetrating procedures such as the deep sclerectomy (DS) have been developed to reduce the incidence of post-operative complications associated with penetrating forms of GFS like trabeculectomy, with intraoperative dissection down to the trabeculo-Descemet window (TDW)—the juxtacanalicular meshwork and inner wall of Schlemm’s canal across which aqueous egresses, followed by removal of a block of deep sclera [4]. The TDW functions as a site of controlled aqueous outflow and also forms an intrascleral space to act as a reservoir [5]. In addition, the natural drainage pathway via the Schlemm’s canal is also augmented [4]. Successful filtering surgeries (blebs) typically have non-adherent sub-conjunctival tissue without scarring and with healthy vasculature, which allows continuous aqueous outflow. A major cause of failure in GFS is fibrosis and scarring of the sub-tenon’s space, which restricts aqueous flow and is caused by fibroblast proliferation and extracellular matrix deposition.

The post-operative evaluation of filtering blebs such as trabeculectomy blebs has traditionally relied on clinical grading systems performed at the slit-lamp, such as the Indiana Bleb Appearance Grading Scale (IBAGS) and the Moorfields Bleb Grading System (MBGS), which document factors associated with surgical success such as bleb area, height, and vascularity. Deep sclerectomy blebs are also assessed clinically at the slit-lamp, although it is uncertain if bleb morphology is correlated with successful IOP reduction given the different aqueous outflow egress pathways compared to in trabeculectomy. This is because while subconjunctival outflow represents an important pathway for egress, augmentation of natural channels such as the Schlemm canal is also an important consequence of non-penetrating glaucoma surgery [4] 

Studies have evaluated the microstructure of filtering blebs using non-contact imaging techniques such as anterior segment Optical Coherence Tomography (AS-OCT) and ultrasound biomicroscopy. These modalities can provide quantitative data on the internal structure of blebs, such as bleb wall thickness, presence of microcysts, and measurements of the internal ostium, bleb cavity, and sub-flap space [6,7,8,9]. The purpose of this cross-sectional study was to optimize and develop a technique for using AS-OCT to image and subsequently quantify the internal bleb microstructure after glaucoma filtration surgery. Specifically, we quantified the dimensions of surgical parameters such as the scleral flap and the trabeculo-Descemet window of filtering blebs following deep sclerectomy surgery.

## 2. Methods

This was a cross-sectional study conducted at the St Paul’s Eye Unit, Royal Liverpool University Hospital, a tertiary referral eye unit in Liverpool, United Kingdom. The study had the approval of the clinical governance department of the Royal Liverpool University Hospital Trust and adhered to the tenets of the Declaration of Helsinki. Consecutive patients attending the glaucoma clinics of the St. Paul’s Eye Unit between November 2022 and February 2023 who had previously undergone deep sclerectomy surgery at least one year prior were recruited. The inclusion criteria were adult patients with a diagnosis of primary open-angle glaucoma, pseudoexfoliative glaucoma, pigment dispersion syndrome glaucoma, or primary angle closure glaucoma. Exclusion criteria were secondary open-angle glaucomas such as uveitic and neovascular glaucoma. All surgeries were performed by consultants and clinical fellows affiliated with the St Paul’s Eye Unit. 

### 2.1. Surgical Technique

The deep sclerectomy surgeries were performed by consultants and clinical fellows affiliated with the St. Paul’s Eye Unit over the years. The main steps shared in common by the surgeons were the creation of a superior fornix-based conjunctival incision and peritomy, the creation of a superficial scleral flap, the creation of a deep scleral flap, exposure of the trabeculo-Descemet window (TDW), excision of the deep scleral flap, suturing of the superficial scleral flap, and conjunctival closure. Intraoperative mitomycin-C was used in a proportion of cases and was performed following the conjunctival peritomy. The variations in dimensions and thickness of the scleral flap and size of the TDW by the different surgeons provided the variations in quantifiable dimensions which were used in the subsequent analysis

### 2.2. Anterior-Segment Optical Coherence Tomography Imaging of Filtering Bleb

**Figure 1:**
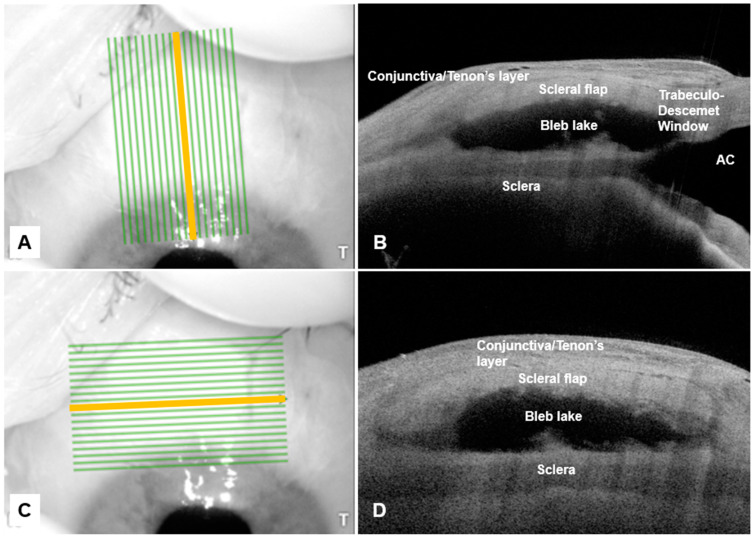
Representative sagittal (**B**) and coronal (**D**) images of a well-functioning deep sclerectomy bleb, with en face oct images in the left column (**A**,**C**). The yellow lines in the left column images represent the sagittal slice overlying the trabeculo-descemet window (TDW) and the coronal slice overlying the mid-point of the flap, which were chosen to produce the adjacent OCT images. Abbreviations: anterior chamber (AC), temporal (T).

The bleb surgical site was first examined with the slit lamp by one of the authors (JT). Anterior-segment Optical coherence tomography of the filtering bleb was then performed by the same author using the Anterion^®^ (Heidelberg Engineering GmbH, Heidelberg, Germany) swept-source OCT device. The Anterion^®^ uses a laser light source with a wavelength of 1300 nm and at 50,000 Hz to obtain B-scans with an axial resolution of 10 microns and transverse resolution of 45 microns. Each scan was performed via the imaging module of the Anterion^®^ device using a standardized raster scan measuring 7.5 mm in width and 12 mm in length and comprising 19 slices. The raster block was first oriented parallel to the long axis of the scleral flap (the sagittal plane in relation to the bleb. (Figure 1) The anterior limit of the image window was positioned just anterior to the limbus at the peripheral superior cornea, which allows the trabeculo-Descemet window within the anterior chamber, iridocorneal angle, and the entire length of the scleral flap to be visualized and captured within the raster slices. The sagittal slice overlying the TDW was identified from the 19 raster slices ) and exported for analysis. The raster block was then oriented perpendicular to the initial sagittal plane to visualize bleb structures in the coronal plane. The anterior limit of the raster block was placed anterior to the TDW to allow the full width of the TDW and scleral flap to be visualized and captured within the raster. The coronal slices overlying the TDW and mid-point of the scleral flap were identified from the 19 raster slices and exported for analysis. 

### 2.3. Image Preprocessing

**Figure 2:**
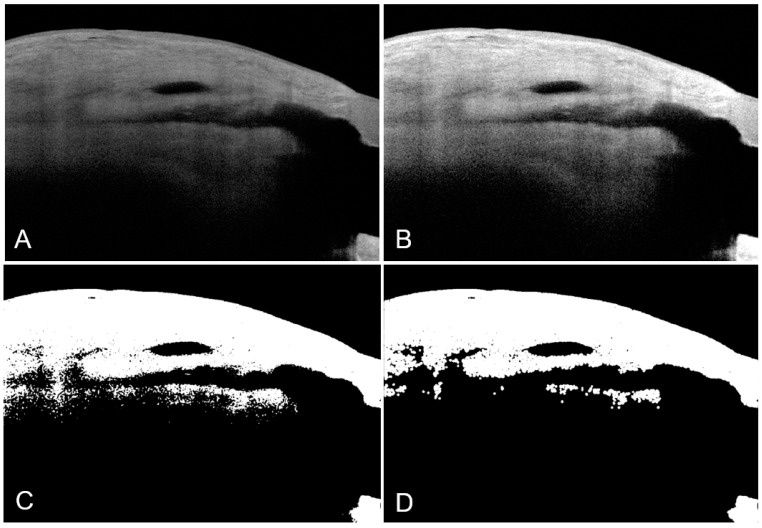
Image processing functions performed on each bleb image—in this representative example using a sagittal trabeculectomy bleb AS-OCT image, to improve visualization of internal bleb microstructure. Original image (**A**), contrast enhancement (**B**), thresholding (**C**), active contouring followed by morphological opening (**D**).

The filtering bleb images were exported to MATLAB for image preprocessing (Mathworks). The following functions were performed on each bleb image sequentially in a standardized manner to improve the visualization of the internal bleb microstructure: conversion to grayscale, contrast enhancement, thresholding, active contouring, and morphological opening (Figure 2). 

### 2.4. Visualisation and Quantification of Surgical Parameters

The dimensions of surgical parameters of interest were then quantified using a measurement tool with the superimposed measurements saved onto a separate file for each image. The dimensions of each surgical parameter were converted from image pixel values to true values using a standardized and verified conversion factor. Table 1 displays the surgical parameters of interest and their standardized anatomical reference points captured on the sagittal and coronal AS-OCT slices.

**Figure 3:**
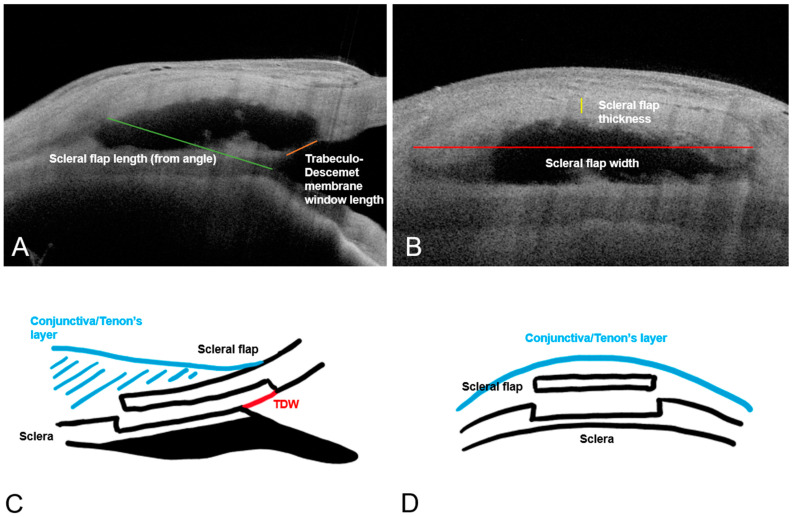
Sagittal (**A**) and coronal (**B**) AS-OCT images of a well-functioning deep sclerectomy bleb with annotated surgical parameters of interest. The relevant anatomical structures are shown in the (**C**) (sagittal) and (**D**) (coronal). TDW: Trabeculo-Descemet window.

The following surgical parameters were visualized on the sagittal slice of each filtering bleb in DS patients: maximal bleb height, scleral flap length, and TDW length (Figure 3). The scleral flap length was measured using the iridocorneal angle as a reference point, as the anterior limit of the scleral flap was indistinct. The scleral flap length is, therefore, not a true flap length given the orientation of the plane of measurement but rather served as a standardized measure across different blebs. The following surgical parameters were obtained from the coronal slice of each filtering bleb: scleral flap width and thickness. 

### 2.5. Definitions of Surgical Success

The surgical outcome of each deep sclerectomy case at the index visit was classified into complete success (CS; IOP ≤ 18 mmHg with no medications), qualified success (QS; IOP ≤ 18 with medications), and failure (F; IOP > 18 mmHg or subsequent filtration surgery procedure performed), as per the World Glaucoma Association consensus on definitions of success 2018 [10].

### 2.6. Statistical Analysis

Descriptive statistics were used to analyze the demographic characteristics of the cohorts. The distributions of quantitative data were first assessed for normality using a D’Agostino and Pearson test of normality, with parametric and non-parametric statistics then applied as appropriate. Analyses were conducted using GraphPad Prism version 9 (GraphPad, La Jolla, CA, USA).

## 3. Results

A total of 107 filtering blebs from 67 patients were included in the study. The mean patient age was 73.0 years (SD 11.2, median 74.1, range 32.6 to 91.0). Sixty patients (89.5%) were of white/Caucasian ethnicity, 4 (6.0%) were Asian, and 3 (4.5%) of afro-Caribbean. The diagnoses were primary open-angle glaucoma (88; 82.2%), primary angle closure glaucoma (9; 8.4%), pigment dispersion glaucoma (4; 3.7%), pseudoexfoliation glaucoma (2; 1.8%), and other/glaucoma not defined (4; 3.7%). The mean best-corrected visual acuity was 0.21 (Snellen equivalent of 20/30. SD 0.23), and the mean deviation was −11.5 dB (SD 7.8) at the index clinic visit. The median post-operative follow-up duration was 6.5 years (IQR 3.1–8.1, SD 4.1 years). The proportion of complete success (CS; IOP ≤ 18 mmHg with no medications), qualified success (QS; IOP ≤ 18 with medications), and failure (F; IOP > 18 mmHg) was 36.8%, 29.1%, and 34.2%, respectively. The mean intraocular pressure and number of medications were 13.3 mmHg (SD 4.9, range 3–27) and 1.1 (SD 1.2, range 0–4), respectively.

### 3.1. Bleb Height, Scleral Flap and Trabeculo-Descemet Window Dimensions and Surgical Outcomes

**Figure 4:**
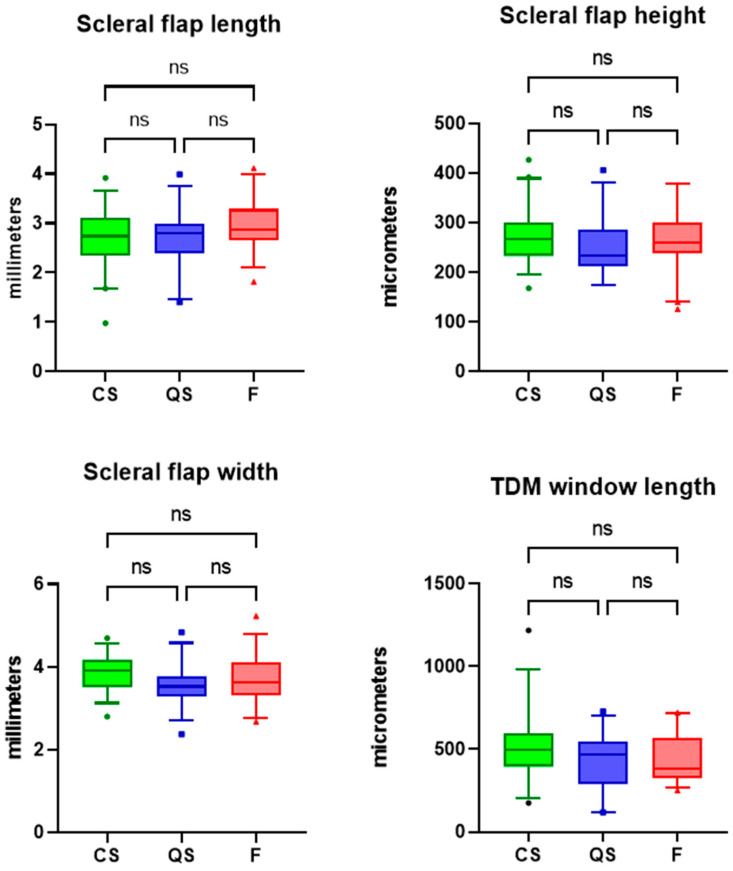
Box and Whisker plots (median, interquartile range, and 5th to 95th percentile) and results of one-way ANOVA of scleral flap dimensions and TDW dimensions across the outcome groups of complete success (CS), qualified success (QS) and failure (F). ns = non-significant.

The maximum bleb height was significantly greater in the complete success blebs compared to the qualified success and failed blebs (1.48 vs. 1.17 vs. 1.10 mm in CS vs. QS vs. F, one-way ANOVA, *p* < 0.0001). Figure 4 and Table 2 display the dimensions of the surgical parameters of interest across the outcome groups of complete success, qualified success, and failure. There was no significant difference in scleral flap length as measured from the iridocorneal angle across the three groups (2.71 vs. 2.73 vs. 2.96 mm in CS vs. QS vs. F, one-way ANOVA, *p* = 0.18). There was no significant difference in scleral flap thickness across the three groups (270.4 vs. 249.9 vs. 267.6 µm in CS vs. QS vs. F, one-way ANOVA, *p* = 0.44). Scleral flap width was also similar across the CS, QS, and F groups (3.85 vs. 3.56 vs. 3.71 mm in CS vs. QS vs. F, *p* = 0.09). The trabeculo-descemet window length was also similar across the three groups (519.0 vs. 432.9 vs. 441.3 µm, one-way ANOVA, *p* = 0.09).

### 3.2. Subanalysis in Deep Sclerectomy Cases Augmented with Intraoperative Mitomycin-C

**Figure 5:**
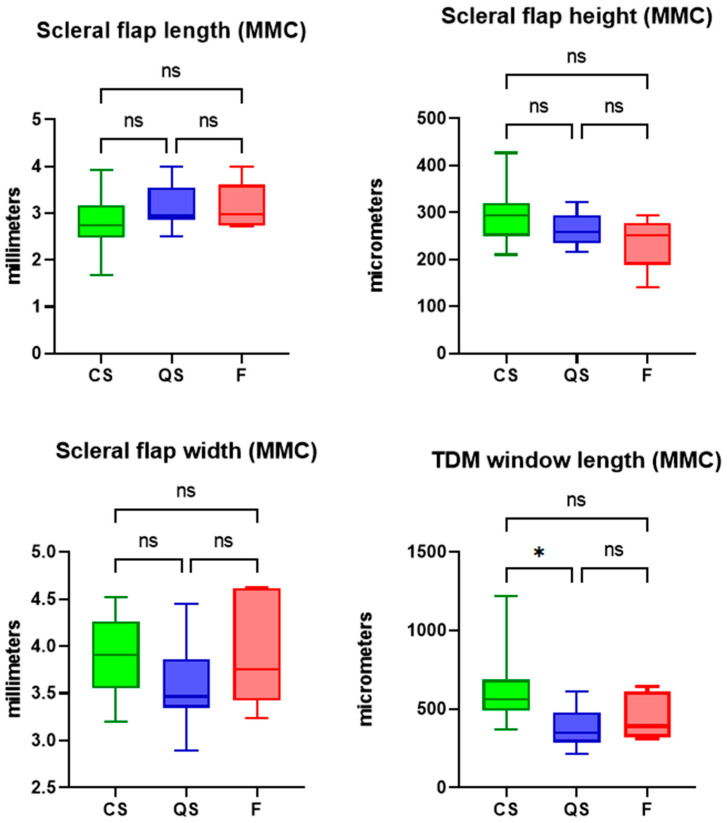
Box and Whisker plots (median, interquartile range, and 5th to 95th percentile) and results of one-way ANOVA of scleral flap dimensions and TDW dimensions across the outcome groups of complete success (CS), qualified success (QS) and failure (F). ns = non-significant. The asterisk denotes statistical significance, i.e., *p* < 0.05.

Of the 107 eyes, 28 (26.2%) underwent scleral application of MMC intraoperatively. Given that intraoperative MMC use may represent a confounder in the results of surgical parameters analysis, we performed a subanalysis of cases of deep sclerectomy augmented with intraoperative Mitomycin-C. In this subcohort, the trabeculo-Descemet window was significantly longer in the complete success compared to the qualified success group (613.7 vs. 378.1 vs. 450.8 µm in CS vs. QS vs. F, *p* = 0.004). There was otherwise similarly no significant difference in scleral flap length, thickness, or width across the three groups (Figure 5).

### 3.3. Post-Operative Duration and Surgical Outcomes

**Figure 6:**
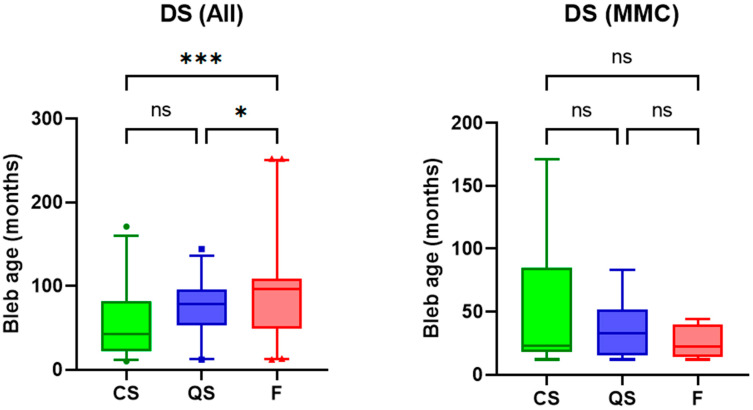
Box and Whisker plots (median, interquartile range, and 5th to 95th percentile) and results of one-way ANOVA of post-operative duration (filtering bleb age) and outcome groups of Complete success (CS), Qualified success (QS), and failure (F). Ns = non-significant. Asterisks denote statistical significance. [i.e., *p* < 0.05 (*), *p* < 0.001 (***)].

The prevalence of failure in glaucoma filtration surgery increases over time [11]. We, therefore, analyzed the relationship between post-operative duration (filtering bleb age) and surgical outcomes. In the overall cohort, filtering blebs with complete success and qualified success had significantly shorter post-operative duration compared to blebs with failure (56.2 vs. 72.1 vs. 98.0 months in CS vs. QS vs. F, one-way ANOVA, *p* = 0.001). This was, however, not statistically significant in cases of deep sclerectomy augmented by intra-operative mitomycin-C only. (Figure 6).

## 4. Discussion

The post-operative evaluation of GFS filtering blebs has traditionally relied on clinical grading systems performed at the slit-lamp, which document factors associated with surgical success such as bleb area, height, and vascularity. In this study, we developed and optimized a technique utilizing swept-source anterior segment-optical coherence tomography to visualize the internal microstructure in the post-operative period of filtering blebs. We then deployed this technique in a cross-sectional cohort of patients who underwent deep sclerectomy surgery with long-term follow-up.

### 4.1. Post-Operative Evaluation of Filtering Blebs

Glaucoma filtration surgery, such as trabeculectomy and deep sclerectomy, is associated with high rates of short- and long-term complications [12]. Long-term complications are often bleb-related, such as bleb fibrosis, leak, and infections or hypotony [13]. Morphological bleb configuration in the post-operative period can influence the planning of follow-up visits in glaucoma patients [14]. Multiple types of clinical bleb grading systems exist, such as the Moorfields Bleb Grading System or Indiana Bleb Appearance Grading Scale, which document factors associated with surgical success such as bleb area, height, and vascularity [6]. These subjective grading systems have varying levels of interobserver agreement on indices and are influenced by the experience of the observer [14]. These subjective grading systems are, however, often used to guide pharmacological and/or surgical intervention in the early post-operative period to decrease the risk of bleb fibrosis and failure. Post-operative evaluation of deep sclerectomy blebs also involves examination of bleb morphology at the slit-lamp; however, it is unclear how bleb morphology correlates with successful IOP reduction. This is because while subconjunctival outflow represents an important pathway for egress, augmentation of natural channels such as the Schlemm canal is also an important consequence of non-penetrating glaucoma surgery [4]. Nevertheless, an objective and quantifiable method for bleb evaluation may also be beneficial in DS surgery.

### 4.2. Swept-Source AS-OCT Technology for More Precise Visualization of Glaucoma Surgeries during the Post-Operative Course

The ANTERION (Heidelberg Engineering, Heidelberg, Germany) and CASIAII (Tomey, Nagoya, Japan) are two commercially available SS-OCT systems specifically designed for the evaluation of the anterior segment. Swept-source technology enables more precise visualization of thicker structures than older OCT modalities, such as spectral-domain OCT, due to greater penetration from longer wavelengths used and higher scan speeds. In our study, we used the precise localization of OCT raster slices to visualize bleb morphology and anatomical details of the drainage outflow channel in a cohort of DS patients. We found that the maximal bleb height was significantly greater in complete success compared to qualified success and failure. We also found that the trabeculo-Descemet window was significantly greater in the complete success compared to the qualified success group in the subcohort of patients who had surgery augmented using intraoperative MMC. To our knowledge, the quantification of these microstructural parameters has not been previously reported and may help surgeons better understand the influence of their surgical technique on outcomes. 

### 4.3. Use of Anterior Segment-OCT in Post-Operative Bleb Evaluation

Previous studies have evaluated the microstructure of glaucoma surgeries using non-contact imaging techniques such as anterior segment optical coherence tomography and ultrasound biomicroscopy [6,15]. These modalities can provide quantitative data on the internal structure of blebs, such as bleb wall thickness, presence of microcysts, and measurements of the internal ostium, bleb cavity, and sub-flap space [6,7,8,9,15]. These parameters are important as they may predict the success or failure of surgery [15]. For instance, Lenzhofer et al. examined 78 eyes of 60 patients post-XEN^®^ gel stent implantation and found that the prevalence of small diffuse cysts was directly associated with lower IOPs, while cystic encapsulation at three months predicted higher surgical failure [16]. Konstantopoulos et al. examined 50 eyes of 50 patients following trabeculectomy, deep-sclerectomy, or no surgery and found that a tall intrascleral lake and a thick conjunctival/tenon’s layer were associated with good post-operative outcomes as defined by intraocular pressure and medication use [17]. Ibarz Barbera et al. used AS-OCT to analyze the morphological evolution of filtering blebs after Preserflo micro shunt implantation and found a progressive horizontal and vertical expansion of the blebs in the sub-Tenon space from baseline to the third month [18]. Gambini et al. similarly characterized Preserflo bleb morphology post-operatively and reported various appearances such as “multiple internal layers” and “microcystic multiform” [19]. In our study, we utilized the high definition provided by swept-source OCT to quantify dimensions of surgical parameters, including the length, width, and thickness of the scleral flap and the length of the TDW. These parameters are important as they are modifiable by the surgeon and also have an effect on aqueous outflow and, therefore, subsequent outcomes. 

The routine use of AS-OCT in the early post-operative period may be of particular clinical relevance following trabeculectomy surgery, especially given the frequency of surveillance and bleb manipulation to manage complications and maximize success during this period. Evaluation of bleb morphology may also be beneficial in deep sclerectomy surgery, such as in capturing the patency of the TDW prior to further laser or surgical intervention, such as a Nd:Yag goniopuncture procedure. The latter has been demonstrated to effectively lower IOP further [20,21] but may be dependent on the patency of the TDW. Other potential use cases for AS-OCT in the early post-operative period include the evaluation of causes of hypotony or, conversely, the evaluation of suspected obstruction of sclerostomy/TDW by the iris or haem which result in elevated IOP. Interventions which may benefit from pre- and post-procedure visualization of bleb internal microstructure include scleral flap suture lysis, the removal of releasable sutures, or bleb massage.

### 4.4. Limitations

We acknowledge several important limitations of our study. Firstly, there are other crucial determinants of surgical success that were not assessed in our analysis, predominantly due to the cross-sectional nature of our study of long follow-up duration. The pre-operative and intra-operative details of surgery were unable to be located in the archived medical records for many patients and had to be excluded from the analysis. These factors include the intraoperative dimensions of the scleral flap, number and type of suture placement on the scleral flap, concentration and duration of intraoperative mitomycin-C use [22], pre-operative intraocular pressure and duration and number of prior topical medications [23], intraoperative complications and early post-operative bleb manipulation [24], which have all been shown to influence post-operative surgical outcomes. Other factors contributing to the heterogeneity of the patient population are also potential confounders of our results, such as ethnicity [25], glaucoma type [26], previous ophthalmic surgery [26], and duration of post-operative follow-up [11], which are known to influence post-operative success and were not controlled for. The focus of our present study was, however, not to analyze the risk factors and outcomes of deep sclerectomy surgery but rather the utility of imaging the microscopic variations in mechanical structural parameters, such as the scleral flap and TDW, which may have a bearing on long-term surgical success. Secondly, the quantification of surgical parameters was performed manually, subjecting these measurements to inaccuracy and bias. We addressed this by keeping a bank of saved images with the measurements superimposed and having a second author verify the measurements. An important limitation of the image analysis was the difficulty in visualizing the parameters of interest in some patients due to poor contrast between these parameters and the surrounding tissues. This was particularly an issue in cases of surgical failure where the flap had likely fibrosed down onto the sclera, making it indistinguishable from the surrounding tissue. The surgical parameters that were indistinguishable had to, therefore, be excluded from the analysis. The amount of data excluded due to poor segmentation represented a minority of the dataset, and we found no systematic difference in outcomes between excluded and included data. Lastly, we used a threshold of 18 mmHg and the criterion of medication use to classify surgical outcomes into complete success, qualified success, and failure in line with recommendations by the World Glaucoma Association consensus on definitions of success. Altering the IOP threshold may, however, change the distribution of our results. We deliberately chose a level of 18 mmHg to reflect the target IOP level we generally aim to achieve in our real-world population of mainly moderate-advanced glaucoma, in line with published outcomes from the Advanced Glaucoma Intervention Study [27]. Considering these limitations, a prospective study using AS-OCT to image trabeculectomy blebs in the post-operative period is required.

## 5. Conclusions

Swept-source anterior-segment OCT can be used to accurately visualize and quantify the surgical parameters which influence aqueous outflow in deep sclerectomy surgery. Our proposed technique of image capture and processing can help surgeons better understand the influence of these parameters on aqueous outflow, which may help improve surgical outcomes.

## Figures and Tables

**Table 1:** Surgical parameters of interest and associated standardized anatomical reference landmarks captured on the sagittal and coronal AS-OCT imaging of DS blebs.

Imaging Plane	Surgical Parameters of Interest	Anatomical Reference Points of Surgical Parameter
Sagittal	Scleral flap length	Posterior edge of scleral flap to iridocorneal angle
	Trabeculo-Descemet window length	Anterior edge of TDW to posterior edge of TDW
Coronal	Scleral flap width	Nasal edge of scleral flap to temporal edge of scleral flap at midpoint of flap
	Scleral flap thickness	Superior edge of scleral flap to inferior edge of scleral flap at midpoint of flap

**Table 2:** Dimensions (mean and standard deviation below) of dimensions of maximal bleb height, surgical flap, and trabeculo-Descemet window length across the outcome groups of complete success (CS), qualified success (QS), and failure (F). *p* value of one-way ANOVA of each parameter is reported.

Parameter	CS	QS	F	*p* Value
Bleb height (mm)	1.48	1.17	1.10	0.001
	0.44	0.44	0.40	
Scleral flap length (mm)	2.71	2.73	2.96	0.178
	0.60	0.54	0.53	
Scleral flap thickness (µm)	270.40	249.90	267.60	0.437
	53.54	53.15	57.26	
Scleral flap width (mm)	3.85	3.56	3.71	0.088
	0.44	0.47	0.58	
Window length (µm)	519.00	432.90	441.30	0.094
	206.40	164.30	146.60	
Scleral flap length, MMC (mm)	2.77	3.11	3.15	0.150
	0.54	0.45	0.51	
Scleral flap thickness, MMC (µm)	298.30	264.40	236.60	0.027
	53.48	34.79	57.81	
Scleral flap width, MMC, (mm)	3.90	3.58	3.97	0.273
	0.40	0.46	0.62	
Window length, MMC (µm)	613.70	378.10	450.80	0.004
	200.20	127.80	152.40	

## Data Availability

Samples of the AS-OCT images of blebs used in this study can be shared upon reasonable request.

## Abbreviations

Anterior-segment Optical Coherence Tomography (AS-OCT), Deep Sclerectomy (DS), Mitomycin-C (MMC), Complete success (CS), Qualified success (QS), Failure (F), Intraocular pressure (IOP).
